# Inflammation and Trauma-Related Psychopathology in Syrian and Iraqi Refugees

**DOI:** 10.3390/bs10040075

**Published:** 2020-04-07

**Authors:** Lana Ruvolo Grasser, Paul Burghardt, Ana M Daugherty, Alireza Amirsadri, Arash Javanbakht

**Affiliations:** 1 Department of Psychiatry and Behavioral Neurosciences, Wayne State University, Detroit, MI 48201, USA; lgrasser@med.wayne.edu (L.R.G.); dy6149@wayne.edu (A.M.D.); aamirsad@med.wayne.edu (A.A.); 2 Nutrition and Food Science, Wayne State University, Detroit, MI 48201, USA; paul.burghardt@wayne.edu; 3 Department of Psychology, Wayne State University, Detroit, MI 48201, USA; 4 Institute of Gerontology, Wayne State University, Detroit, MI 48201, USA

**Keywords:** refugees, inflammation, trauma, psychopathology, anxiety, PTSD, depression

## Abstract

Refugees experience high rates of post-traumatic stress disorder (PTSD), anxiety, and depression due to exposure to civilian war trauma and forced migration. Inflammatory products may offer viable biological indicators of trauma-related psychopathology in this cohort, promoting rapid and objective assessment of psychopathology. Incoming Syrian and Iraqi refugees (n = 36) ages 18–65 completed self-report measures of PTSD, anxiety, and depression and provided saliva samples during an assessment at a primary care clinic within the first month of resettlement in the United States. Interleukin 1β (IL-1β) and C-reactive protein (CRP) differentially correlated with symptom severity by domain, and there was a non-significant trend for sex moderating the relation between inflammation and PTSD symptoms. Our findings show unique relations between trauma-related psychopathology and inflammation. There is a need for further research in diverse ethnic cohorts with differential trauma exposures for inflammation to be considered a biological indicator of psychopathology.

## 1. Introduction

An estimated 70.4% of the global population is exposed to trauma in a lifetime [1], however not all individuals exposed develop a trauma-related psychological disorder; only approximately 6.8% develop post-traumatic stress disorder (PTSD) [2]. An enduring aim in trauma research is understanding the factors that may predispose an individual to develop a trauma-related disorder. Recently, inflammatory response has been implicated as a key component in the pathophysiology of trauma [3]. 

During trauma exposure, physiological changes leading to sympathoexcitation and activation of motor pathways are beneficial for mounting appropriate responses to danger. Activation of the sympathetic adrenomedullary axis (SAM) is a key component of the stress response following a traumatic event or reminders of that event [4,5]. The SAM axis triggers the release of catecholamines epinephrine and norepinephrine, alternatively known as adrenaline and noradrenaline [6]. Catecholamine secretion in turn leads to the production and secretion of pro-inflammatory cytokines and acute phase proteins to drive immune activity via induction of NF-κB [7]. Acute activation of this system is adaptive, however chronic activation is maladaptive [8]. After the traumatic event has ceased, these physiological responses may become sensitized and maladaptive such that stress and trauma-related stimuli can elicit them in the absence of danger [9]. For example, individuals may show attention bias towards threatening stimuli and become hypervigilant and aroused [9]. Chronic activation of the SAM and immune system results in diminished vaccine responses [10], exacerbated viral and bacterial pathogenesis [11], stunted wound healing [12], alteration in autoimmune diseases, and exacerbated psychiatric susceptibility and severity [13]. As such, trauma and psychological stress increase likelihood of disease, circulating levels of pro-inflammatory cytokines, and acute phase immune proteins. 

Some pro-inflammatory cytokines, such as interleukin 1β (IL-1β), have been shown to be elevated in individuals exposed to combat trauma, abuse, or man-made accidents as compared to non-exposed healthy controls [14]. Previous research has also linked increased circulating levels of IL-1β with trauma exposure, childhood maltreatment, and PTSD [14,15,16,17,18]. Two studies have shown evidence for elevated concentrations of IL-1β in individuals with panic disorder [19,20]. cDNA array chip analysis indicates downregulation of IL-18 related genes in individuals with PTSD [21], and genotype by trauma interactions have shown transcripts for IL-18 to be positively correlated with post-traumatic stress symptom severity [22]. Taken together, elevated inflammatory state has been shown to increase the risk of developing PTSD following a traumatic event [23,24], and pro-inflammatory cytokine expression may mediate the relation between early life stress and adulthood psychiatric and physical disorders [25].

In addition to increased pro-inflammatory cytokine response, elevated levels of acute phase proteins, namely C-reactive protein (CRP) [3] may be an indicator for trauma-related pathophysiology [20,26,27]. CRP concentration increases during infection and trauma, and minor elevations in CRP may be indicators of future cardiovascular disease [28]. CRP may also represent a risk factor for PTSD, as higher pre-deployment concentrations of CRP predict increased risk for PTSD at post-deployment in a sample of military personnel [29]. Not only does CRP predict risk for PTSD, but also severity of symptoms and fear-potentiated startle, a behavioral measure of trauma-related symptomatology and pathology [30,31]. Yet similar to pro-inflammatory interleukin expression, other research studies show no relation between PTSD symptoms and circulating CRP [32]; one study reported elevated inflammatory cytokines, including IL-1β, were associated with post-traumatic pathology, whereas CRP was not [33]. Levels of CRP may be elevated in patients with generalized anxiety disorder (GAD) compared to controls, and concentrations may increase over time for patients with agoraphobia [34,35,36]. Lower levels of CRP and serum amyloid A (another acute phase protein) have previously been reported in Iraqi refugees with PTSD compared to those without [37]. This may suggest differential ethnic or racial inflammatory responses to trauma, which is an underexplored area of research. Inconsistent replication of a relation between inflammation response and PTSD symptoms or diagnosis can be attributed to a number of study design features but perhaps most important to consider is the representation of meaningful demographic factors such as race or ethnicity, and sex [3]. 

Sex differences in pro-inflammatory response following trauma may partially account for individual differences in the development of trauma-related psychopathology. Women with fatigue have higher levels of CRP than men with fatigue [38], and experimentally induced inflammation is more strongly associated with socioemotional changes in women compared to men [39]. One study of 1294 middle aged Finnish adults reported higher levels of CRP in women compared to men [40,41], as did another study of 2,749 Caucasian and Black women [42], and a third more diverse cohort study of 6814 adults [43]. A small cohort gene expression study found that consistent with these observations, under-expression of genes on monocytes—immune cells that both initiate and respond to inflammatory signals—was observed in men with a diagnosis of PTSD, whereas women with PTSD displayed highly-variable expression [44]. All together, these data support a hypothesis that women may have higher inflammatory states compared to men, and that the relation between trauma exposure and related psychopathology is greater in women than men. 

Trauma is high among Syrian and Iraqi refugees [45,46,47]. However, little attention has been paid to this unique population in research on trauma and trauma-related physiology, psychopathology, and treatment. All may show variation from traditional research cohorts due to potential underlying genetic differences and environmental factors. Our present study examines the relation between inflammation and psychopathology in trauma-exposed Syrian and Iraqi refugees. Here we evaluate complementary and quantifiable indicators of inflammatory response measured from saliva samples: CRP, IL-1β, and IL-18. Based on previous work [3], we hypothesized that elevation in all three indicators would be associated with greater symptom severity of PTSD, anxiety, and depression. We also hypothesized that women would have elevated inflammation as compared to men, and that the relationship between inflammation and symptom severity would be greater for women compared to men. This cohort provides the benefit of homogenous trauma exposure—civilian war trauma and migratory stress with exposure commencing and ending within a similar timeframe for all individuals—and all data having been collected within one month following migration. 

## 2. Materials and Methods

Participants were 36 adult refugees (20 females, 16 males) from Syria (25) and Iraqi (8) (3 did not report origin) who resettled in southeast Michigan between June 2016 and May 2017, part of a larger cohort study of refugee mental health [45,46,47]. All participants originated from Syria or Iraq during the same time period and were refugees of war. Participants were recruited at primary care clinics during mandatory physical health screenings that occur within the first month of resettlement in the United States. Physicians informed individuals of the opportunity to take part in a voluntary research study following the physical health assessment. Those who were interested were introduced by the physician to the research team and were provided with an informed consent form to review and sign if they chose to participate in the study. All study procedures received Institutional Review Board approval from Wayne State University—IRB #012416B3F. 

A demographic questionnaire was used to gather information regarding age, sex, body mass index (BMI), any substance use, medical conditions, medication use, psychiatric conditions, and psychiatric treatment. Self-report questionnaires were then administered in both Arabic and English. Adults completed the PTSD Checklist for Civilians (PCL-C for Diagnostic and Statistical Manual of Mental Disorders version IV (DSM IV)) [48,49] as a measure of post-traumatic stress symptoms and the Hopkins Symptoms Checklist (HSCL-25) [50] as a measure of anxiety and depression. Details of scoring methods have previously been reported in a separate article [45,46]. Briefly, a probable diagnosis for PTSD based on the PCL-C is determined by meeting above-threshold scores across criteria B, C, D, and E as defined by the DSM-IV. We have previously used a cutoff of 40 for presence of probable PTSD based on total sum scores [45,46]. For the HSCL-25, a score of 1.75 or higher for the average of the first 10 items is indicative of probable anxiety; a score of 1.75 of higher for the average of the last 15 items is indicative of probable depression. Demographic data are presented in Table 1. 

Following completion of demographic forms and questionnaires, 1mL saliva samples were obtained by passive drool into a 2mL collection tube over a timed 60 s period at rest. Participants had not eaten nor drank at least 30 min prior to collection, and saliva production was not stimulated by any task or action. Samples were collected mid-day on average to avoid the peak and nadir of production [51]. Following collection, samples were frozen at −80 °C until assay. Levels of CRP, IL-1β, and IL-18 were determined using an AlphaLISA kit (Perkin Elmer, Waltham, Massachusetts, United States) in single batches to eliminate inter-assay variability. 

All statistical tests were performed in IBM SPSS v.25 [52]. Standard data screening for normality was conducted and satisfactorily met. Missing data occurred in 2 cases for IL-1β, 4 cases for IL-18, 1 case for the PCL, 6 cases for the HSCL-anxiety, and 10 cases for the HSCL-depression; resulting in 5.6% of IL-1β, 11.1% of IL-18, 2.8% of PCL, 16.7% of HSCL-anxiety, and 27.8% of HSCL-depression measures missing at random (Little’s χ^2^ = 32.83), and missing values were replaced with the sample mean, which introduces minimal bias in this application and is preferred to listwise deletion [53]. In this manner, all analyses were conducted with the total available sample (n = 36) and repeated for an assessment of similar effect sizes in the sample with complete data (n = 22). 

In a 3-level repeated measure general linear model (GLM), greater symptom severity of PTSD, anxiety, and depression were predicted by inflammation indicators (IL-1β, IL-18, and CRP). This model design takes into account correlations and comorbidities of reported symptoms across diagnostic categories when evaluating total and unique effects of inflammation indicators. To allow this comparison across scales, the PCL, HSCL-anxiety, and HSCL-depression were converted to standardized z-scores. A significant interaction term would indicate a differential effect of inflammation across psychopathologies. 

To evaluate significant omnibus effects, the correlation of the inflammation indicator and symptom severity was compared between symptom domains with Steiger’s Z*, controlling for the correlation between domains. 

To test the second hypothesis that women had elevated inflammation as compared to men, and the relation between inflammation and symptoms severity differed by sex, we created a composite variable from the three inflammation indicators. The three indicators were submitted to a principle components analysis to extract a single component, representing a weighted composite inflammation score. The use of a composite pro-inflammatory score has previously been used to investigate the relation between IL-1β, IL-6, TNF-α, INF-γ, CRP, and combat exposed males with PTSD compare to combat exposed males without PTSD [54]. Other studies have chosen to sum z-score transformed concentrations for each marker [55,56,57]. We performed analyses deriving composite pro-inflammatory scores both ways, and yielded the same results regardless of computational method. In a hierarchical regression, inflammation score was tested as a predictor of symptom severity with sex as a moderator. With the addition of the sex × inflammation interaction, significant increase in R^2^ and a significant b-weight were interpreted as evidence of moderation. Due to the restrictions from sample size, each symptom domain was tested separately, and a Bonferroni correction for multiple comparisons was made (α’ = 0.02). 

## 3. Results

A total of 36.1% (n = 13) of individuals screened positive for probable PTSD based on total severity scores from the PCL-C with a threshold score of 40. Almost half (n = 17, 47.2%) screened positive for anxiety and 1/3^rd^ (n = 12, 33.3%) screened positive for depression based on the HSCL. Since diagnoses were not made by clinician interview, and in line with the NIH Research Domain Criteria to assess psychopathology on a continuum, we did not group analyses based on diagnosis henceforth. 

While a medical evaluation was not conducted by the research team, chart review as consented to by participants yielded BMI data. Some participants reported medication use (13; 36.1%) and of these, 5 (14% of the total sample) were using an anti-inflammatory medication. Medication use was predominantly for the purpose of alleviating headaches (NSAIDs) and for birth control. No participants reported a serious medical condition. No participants were taking psychiatric medications or were in psychotherapy, and no participants in this sample reported any substance use (including smoking). We did not find any effect of medication on levels of inflammation or relations between inflammation and symptoms (all p’*s* > 0.07), and none of these individuals were outliers in reported psychiatric symptoms. Previous work suggests that age and BMI can also affect inflammatory state. We did not find age or BMI to be predictors of inflammation or symptoms (all p’s > 0.12). It should be noted, however, that these tests were likely underpowered with this sample size.

Intra-assay coefficients of variation for IL-1β, IL-18, and CRP were as follows: 6.82%, 6.21%, and 4.44%. All assays were run in a single-batch (duplicates on a 384 well plate). 

### 3.1. Relation between Inflammation and Psychiatric Symptom Severity

**Figure 1:**
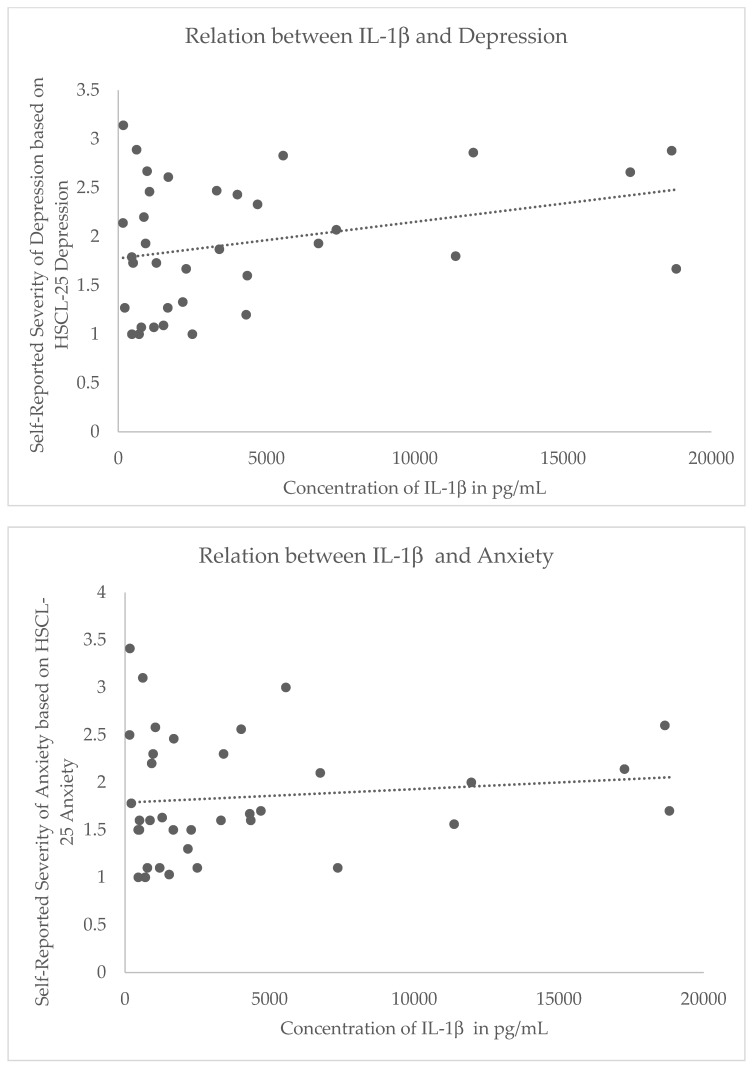

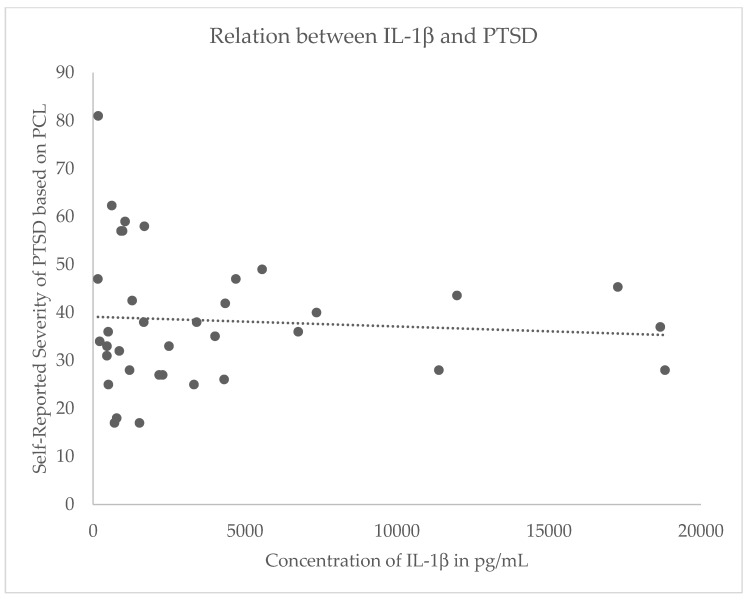
Steiger’s Z* revealed differential relations between IL-1β and depressive symptoms (top), IL-1β and anxiety symptoms (middle), and IL-1β and post-traumatic stress symptoms (bottom). While these relations were not significant, the difference in correlation coefficients was significant, indicating that there is a differential relation between inflammation and trauma-related psychopathology. Y axes reflect self-reported symptom severity scores for the Hopkins Symptoms Checklist (HSCL)-depression index (range: 0–4) (top), HSCL-anxiety index (range: 0–4) (middle), and posttraumatic stress disorder (PTSD) Checklist for Civilians (PCL) for DSM IV (range: 17–85) (bottom).

In a repeated-measure GLM, we found no evidence for a main effect of IL-1β (F(1,32) = 0.86, p = 0.36, partial η^2^ = 0.03), IL-18 (F(1,32) = 0.70, p = 0.41, partial η^2^ = 0.02), or CRP (F(1,32) = 0.93, p = 0.34, partial η^2^ = 0.03) on symptom severity, averaged across domains. However, the multivariate tests demonstrate significant interaction terms that indicate IL-1β (F(2,31) = 5.56, p < 0.01, partial η^2^ = 0.26) and CRP (F(2,32) = 4.27, p = 0.02, partial η^2^ = 0.21), but not IL-18 (p = 0.91; partial η^2^ = 0.006), differentially correlated with symptom severity by domain (Figure 1). Repeating the analysis with listwise deletion of cases with missing data, the pattern of results was similar: there was no evidence for a main effect of IL-1β (F(1,18) = 1.11, p = 0.31, partial η^2^ = 0.06), IL-18 (F(1,18) = 0.07, p = 0.80, partial η^2^ = 0.004), or CRP (F(1,18) = 0.44, p = 0.52, partial η^2^ = 0.02) on symptom severity, averaged across domains. For the multivariate tests, there was no evidence of IL-1β (F(2,17) = 0.36, p = 0.70, partial η^2^ = 0.04) or IL-18 (F(2,17) = 0.92, p = 0.42, partial η^2^ = 0.10) differentially correlating with symptom severity by domain; the tests failed to reach significance with reduced power. However, the results were similar for CRP (F(2,17) = 3.51, p = 0.05, partial η^2^ = 0.29). 

### 3.2. Differential Relations between Inflammation and Symptom Severity by Domain

**Figure 2:**
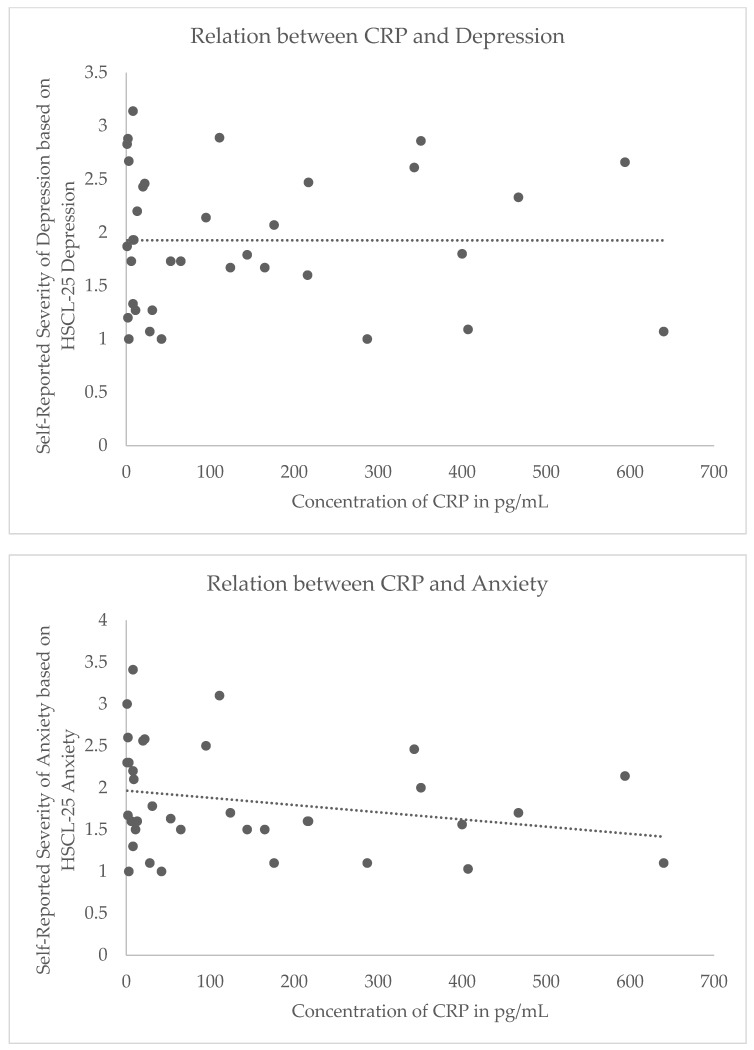
Steiger’s Z* revealed differential relations between C-reactive protein (CRP) and depressive symptoms (top) and CRP and anxiety symptoms (bottom), where no relation exists between the former and a slightly negative relation appears between the latter. While these relations were not significant, the difference in correlation coefficients was significant, indicating that there is a differential relation between inflammation and trauma-related psychopathology. All Y axes reflect concentration of CRP in pg/mL. X axes reflect self-reported symptom severity scores for the HSCL-depression index (range: 0–4) (top) and HSCL-anxiety index (range: 0–4) (bottom).

Following the evidence of a differential effect of inflammation on symptom severity between domains, post-hoc comparisons were made. Whereas elevated IL-1β showed a non-significant inverse relation with PTSD (r(34) = −0.11, p = 0.52), IL-1β showed a non-significant direct relation with depression (r(34) = 0.28, p = 0.10; Z* =−3.12, p < 0.01) and a non-significant positive trend with anxiety (r(34) = 0.07, p = 0.67; Z* = −2.09, p = 0.04). In addition, CRP was more negatively (yet not significantly) correlated with anxiety (r(34) = −0.28, p = 0.11) as compared to depression (r(34) = −0.03, p = 0.10; Z* = −2.52, p = 0.01; Figure 2). All other comparisons were not significantly different (all p’s > 0.05). It is noteworthy that although the effects of these inflammation indicators differed between symptom domains, the magnitude of bivariate correlation with symptom severity in any single domain did not reach nominal significance in this sample. Repeating the analysis with listwise deletion of cases with missing data, the pattern of results for CRP were similar: CRP was negatively, yet non-significantly, correlated with anxiety (r(20) = −0.23, p = 0.31) while CRP was positively, yet non-significantly correlated with depression (r(20) = 0.23, p = 0.31; Z* = −2.449, p = 0.01). 

### 3.3. Sex Differences in Inflammation, and Relation with Symptom Severity

**Figure 3:**
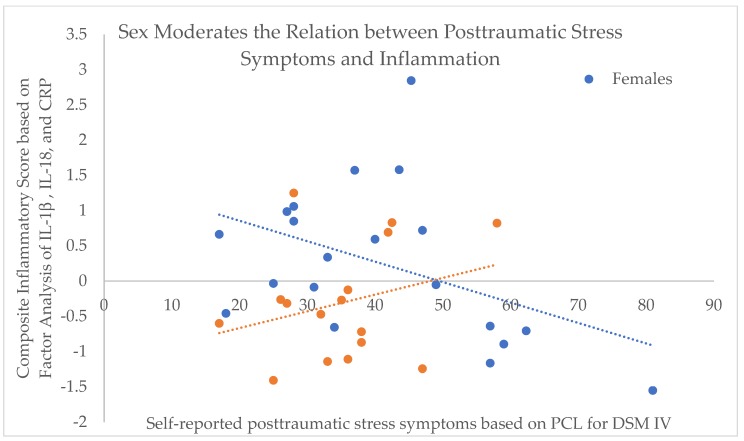
Moderation analysis provides evidence towards a potential role of sex on the relation between post-traumatic stress symptoms and pro-inflammatory variables (IL-1β, IL-18, and CRP).

A composite variable for inflammation was created using principle components analysis, and the resulting factor score was then used to test the hypothesis of sex-related differences in inflammation response and correlated symptom severity. An independent samples t-test indicated that inflammation did not differ by self-reported biological sex, t(34) = −1.71, p = 0.10. Additionally, reported severity of anxiety, depression, and PTSD symptoms did not differ by sex (all t’s < −0.80, p’s > 0.06). There was no evidence of sex moderating the relation between inflammation and symptom severity in anxiety (change in R^2^ = 0.05, F(1,32) = 1.87) or depression (change in R^2^ = 0.01, F(1,32) = 0.44). There was a non-significant trend for sex moderating the relation between inflammation and PTSD symptom severity (b = −10.09, p = 0.05): change in R^2^ = 0.1023, F(1,32) = 4.13, p = 0.05; model F(3,32) = 2.79, p = 0.06 (Figure 3). Repeating the analysis with listwise deletion of cases with missing data, there were no significant moderating effects of sex on the relation between PTSD (change in R^2^ = 0.0067, F(1,21) = 0.50, p = 0.50), anxiety (change in R^2^ = 0.019, F(1,21) = 2.86, p = 0.11), or depression (change in R^2^ = 0.0069, F(1,21) = 0.60, p = 0.45) and inflammation. With reduced power, the tests failed to reach significance. 

## 4. Discussion

In this study, we assessed the relation between inflammation and PTSD, anxiety, and depression symptoms in Syrian and Iraqi refugees. All of these conditions have been previously shown to have positive relations with pro-inflammatory cytokines. However, the evidence derived from our small, homogenous cohort indicates that there may be differential inflammatory mechanisms involved in different consequences of stress and trauma exposure (being anxiety, depression, and PTSD). Most prior studies have examined inflammation in relation to only one of these symptoms categories, often times PTSD, and have ignored the comorbidity of trauma-related psychopathology. The results from our present study should be interpreted with care given the small sample size and considered preliminary/exploratory. This evidence calls for greater study of inflammatory profiles across psychological domains in traumatized populations.

Our data from a unique cohort of physically healthy Syrian and Iraqi refugees (relatively young, similar trauma, timing of exposure and stress of migration, and timing of data collection within the first month of their arrival in the host country) showed a negative trend for IL-1β predicting PTSD, where lower concentrations of IL-1β were associated with greater severity of post-traumatic stress symptoms. Conversely, we observed a positive trend for greater IL-1β expression predicting higher anxiety and depression symptoms severity. These results replicate previous findings of increased inflammation in patients with depression; indeed, IL-1β is known to be one of the most reliable biomarkers of inflammation in these patients [58]. While there was no statistically significant effect of IL-1β expression on symptom severity, the magnitude and the direction of the effect significantly differed between symptom domains, and we plan to evaluate this further in the future with a larger cohort. 

Similarly, CRP also showed a negative relation to anxiety and depressive symptom severity. Neither bivariate effect was significant, however there was a larger effect of CRP on anxiety as compared to depression. Yet this effect trended in the negative direction; lower basal concentration of CRP predicted greater anxiety symptoms severity. Unlike CRP and IL-1β, there were no significant findings or trends regarding IL-18 and psychological symptoms. 

Based upon this admixture of evidence, it is unlikely that the pathophysiology of inflammation is universal in the development of different trauma-related disorders [3,42,59,60]. Additional factors likely modify the effects observed; for example, there was marginal evidence of sex moderating the relation between PTSD symptoms and inflammation, and future research should place a greater focus on sex as a biological variable potentially driving relations between inflammation and psychological symptoms as well as possible diagnoses. Trauma type may also have a role, and there may be differences between refugee populations exposed to war trauma compared to other groups more commonly assessed in the literature, including victims of motor vehicle accidents, assault/abuse, and combat veterans. Type of trauma exposure is critical, as chronic, interpersonal traumas may yield more passive, blunted behavioral and sympathetic responses, while acute, non-interpersonal traumas may yield more active, activating behavioral and sympathetic responses [9]. Finally, uniqueness of our findings in a novel subject population with dominant literature focused on Caucasian and Black/African American racial backgrounds signifies the importance of larger future studies of inflammation and trauma in other ethnic and racial groups; indeed some of the relations we found between psychopathology and inflammatory variables were more akin to findings from a sample of Iraqi individuals [37]. This could then direct the field towards examination of potential genetic underpinnings of such differences. 

Moreover, studies that use a single symptom domain, or an assessment that that is contaminated by comorbid symptoms, may have less sensitivity to the effect of inflammation. In other words, if there are opposing effects on IL-1β between two different symptom domains, then a combined assessment would likely produce a null effect. An advantage of our study is that we evaluated multiple symptom categories in relation to inflammation; creating a combined psychopathology severity variable, we determined that there were no main effects of inflammation on this combined variable, however there were differences based on distinct domains of psychopathology. These findings from a small sample may provide further insight into the discrepant results in the current body of literature and speak to the need for standardized and specific assessment measures, taking into account variant psychopathology that may emerge following trauma exposure in the context of diverse populations. Our present study provides the groundwork for multidomain testing and analyses across domains.

Our findings are limited by a relatively small sample size, which was due to our limited ability and time available for data collection within a primary care setting, as well as changes in the US policies in accepting refugees. Psychopathology was based on self-report questionnaire data and not clinical interview. Previous research found that depression was associated with raised IL-1β levels [61]; the association between PTSD and IL-1β levels found in this study could be confounded by depression. Further research is required to evaluate the effect of pharmacotherapy to reduce IL-1β levels in refugees as previous research found that antidepressants were able to reduce IL-1β levels [62]. Due to the research environment—within primary care offices following physical health examinations required of by all newly arriving refugees—conducting a full trauma interview was not possible. 

If inflammatory state can be well-established in terms of its relationship to trauma-related disorders including PTSD, anxiety, and depression, then there may be preventative or therapeutic applications. Identifying causation was not a goal of this study, nor would it be possible given the present design. As indicated, there may be a relation between inflammation and trauma-related psychopathology. Treating one may help improve the other. Pre-existing heighted inflammation may be a risk factor for greater pathology following trauma exposure, and additionally heighted inflammation following trauma may predict those at greater risk for psychopathology, and therefore intervention could be implemented immediately following exposure and prior to onset of psychopathology. Our findings emphasize that further work is necessary to investigate inflammatory correlates of psychopathology by sex, in specific ethnic cohorts, and based on comorbidities. 

## Figures and Tables

**Table 1:** Descriptive statistics for sample. Units for inflammatory variables are pg/mL. Values for PTSD, anxiety, and depression denote total severity scores on self-report psychological questionnaires. Mean severity scores for anxiety and depression were above the probable diagnostic threshold of 1.75 each; mean severity score for PTSD was below the probable diagnostic threshold of 40.

	Age	Sex	IL1β	CRP	IL18	PTSD	Anxiety	Depression
**n**	36	36	36	36	36	36	36	36
**Mean**	36.639	0.556	4017.320	140.929	9952.472	38.327	1.845	1.928
**Std. Deviation**	10.965	0.504	5223.862	180.076	8068.229	14.020	0.633	0.646
**Minimum**	19.000	0.000	161.954	1.090	1338.796	17.000	1.000	1.000
**Maximum**	65.000	1.000	18820.207	639.670	40018.593	81.000	3.412	3.144
